# Toward a Closed Loop, Integrated Biocompatible Biopolymer Wound Dressing Patch for Detection and Prevention of Chronic Wound Infections

**DOI:** 10.3389/fbioe.2020.01039

**Published:** 2020-09-01

**Authors:** Andrew C. Ward, Prachi Dubey, Pooja Basnett, Granit Lika, Gwenyth Newman, Damion K. Corrigan, Christopher Russell, Jongrae Kim, Samit Chakrabarty, Patricia Connolly, Ipsita Roy

**Affiliations:** ^1^Department of Biomedical Engineering, Faculty of Engineering, University of Strathclyde, Glasgow, United Kingdom; ^2^School of Life Sciences, College of Liberal Arts and Sciences, University of Westminster, London, United Kingdom; ^3^Vivisco Pvt Ltd, London, United Kingdom; ^4^School of Mechanical Engineering, Faculty of Engineering, University of Leeds, Leeds, United Kingdom; ^5^School of Biomedical Sciences, Faculty of Biological Sciences, University of Leeds, Leeds, United Kingdom; ^6^Department of Materials Science and Engineering, Faculty of Engineering, The University of Sheffield, Sheffield, United Kingdom

**Keywords:** wound dressing, biopolymer, pyocyanin, *Pseudomonas aeruginosa*, graphene, Polyhydroxyalkanoates, artificial intelligence, electrochemical

## Abstract

Chronic wound infections represent a significant burden to healthcare providers globally. Often, chronic wound healing is impeded by the presence of infection within the wound or wound bed. This can result in an increased healing time, healthcare cost and poor patient outcomes. Thus, there is a need for dressings that help the wound heal, in combination with early detection of wound infections to support prompt treatment. In this study, we demonstrate a novel, biocompatible wound dressing material, based on Polyhydroxyalkanoates, doped with graphene platelets, which can be used as an electrochemical sensing substrate for the detection of a common wound pathogen, *Pseudomonas aeruginosa*. Through the detection of the redox active secondary metabolite, pyocyanin, we demonstrate that a dressing can be produced that will detect the presence of pyocyanin across clinically relevant concentrations. Furthermore, we show that this sensor can be used to identify the presence of pyocyanin in a culture of *P. aeruginosa*. Overall, the sensor substrate presented in this paper represents the first step toward a new dressing with the capacity to promote wound healing, detect the presence of infection and release antimicrobial drugs, on demand, to optimized healing.

## Introduction

Chronic wound infections can have life changing consequences to patients and represent a significant burden to healthcare providers such as the NHS. Infections delay wound healing and can result in a worsening of the patient’s condition. A wound is considered to be chronic if it does not show evidence of progression through the normal healing stages within a timely manner, usually considered to be 30 days (Frykberg and Banks, 2015). The most common chronic wounds are diabetic ulcers, vascular ulcers and pressure ulcers. A number of strategies have been explored to improve chronic wound outcomes. Tissue engineering approaches, such as the use of stem cells, modulating the immune response, and the use of scaffolds, cell therapies or growth factors are widely reported (Rezaie et al., 2019). Evidence based decision systems have also been developed to progress from decision based support systems (Schaarup et al., 2018) and a large field of research is devoted to the point of care monitoring and analysis of chronic wounds (Sheets et al., 2016).

Chronic wounds are at a great risk of infection due to the prolonged loss of the protective skin barrier providing an attractive climate for bacterial colonization. *Pseudomonas aeruginosa* is one of the most common bacteria that infect chronic wounds (Serra et al., 2015). For example, in one study, *P. aeruginosa* was isolated in 52.2% of chronic leg ulcers and ulcers infected by *P. aeruginosa* were shown to cover a significantly larger area than those in which the bacteria was not present (Gjødsbøl et al., 2006). Furthermore, *P. aeruginosa* has been shown to infect deeper regions of the wound bed, often forming resistant biofilms (Kirketerp-Møller et al., 2008; Serra et al., 2015). In this way *P. aeruginosa* can develop a serious and persistent infection within chronic wounds, greatly contributing to the likelihood of resorting to more severe treatments, such as amputation. Additionally, *P. aeruginosa* is widely associated with burn wounds, where it is responsible for high rates of morbidity and mortality (Bodey et al., 1983; McManus et al., 1985; Sharma et al., 2006; Öncül et al., 2014).

Due to the persistent nature of *P. aeruginosa* infections, the ability to detect the presence of the bacteria would allow for early diagnosis and application of an appropriate treatment plan before the infection becomes too widespread. Wound pH is widely recognized as a key factor in wound healing and has been used as a basis for the development biosensors to detect infection and a number of previous investigators have used this as a basis for infection detection (Songkakul et al., 2019; Kuo et al., 2020). Some have also included closed loop release of antimicrobials in the device design (Mostafalu et al., 2018; Rivero et al., 2020). Humidity and analysis of the microenvironment within the wound have also been used as sensor strategies to assess the progress of wound healing and potentially predict the onset of infection (McColl et al., 2007, 2009). Recent reviews of the state-of-the-art in smart wound dressings can be found in Scalamandré and Bogie (2020), and McLister et al. (2017).

Phenazines are redox-active, highly diffusible and soluble compounds that are produced by a range of bacteria as secondary metabolites during the stationary phase of growth (Mavrodi et al., 2006). In particular, the phenazine, pyocyanin is produced by 96–98% of *P. aeruginosa* strains (Reyes et al., 1981). Pyocyanin production is density-dependent; regulated through QS signals, with a variety of roles in the pathogenicity (Lau et al., 2004) and phenotypic characteristics of the bacteria (Dietrich et al., 2008). The redox-active properties of pyocyanin are widely reported to enhance bacterial pathogenicity, but it also provides an opportunity for electrochemical detection of *P. aeruginosa* (Wang and Newman, 2008; Sharp et al., 2010; Bellin et al., 2014; Ward et al., 2014; Alatraktchi et al., 2016; Sismaet et al., 2016).

The biopolymer used in this study is a medium chain length Polyhydroxyalkanoate (MCL-PHA) which belongs to a family of natural biopolyesters of 3-, 4-, 5-, and 6-hydroxyalkanoic acids. PHAs are synthesized by a wide range of bacteria such as *Bacillus cereus*, *Pseudomonas putida*, *Pseudomonas oleovorans* and act as energy reserves under nutrient limiting conditions. These limiting conditions hinder cell growth and division and switch on the biosynthetic pathway of the PHAs (Valappil et al., 2007, 2008; Hyakutake et al., 2011). Hence, bacteria including *Cupriavidus necator*, *Alcaligenes latus*, *Pseudomonas mendocina* accumulate these polyesters as insoluble inclusions in the cytoplasm. These water insoluble polymers are biodegradable, thermoplastic and exhibit high molecular weight (Byrom, 1994). PHAs can be divided into two main classes: Short chain length PHAs (SCL-PHAs), that have monomers consisting of 3–5 carbons such as poly(3-hydroxybutyrate), P(3HB); poly-4-hydroxybutyrate, P(4HB); and poly(3-hydroxybutyrate-co-3-hydroxyvalerate), P(3HB-co-3HV), are partially crystalline, and thermoplastic in nature. They generally lack toughness, except for P(4HB) and hence are brittle polymers and have high melting points. MCL-PHAs have monomers consisting of 6–14 carbons such as polyhydroxyundecenoate, PHU and Poly(3-hydroxyoctanoate), P(3HO) (Dufresne and Vincendon, 2000; Rai et al., 2011) and these polymers are elastomeric in nature with low crystallinity, low tensile strength, low melting point and high elongation at break (Nomura and Taguchi, 2007). The degradation products of PHAs (hydroxyl acids) have almost no cytotoxicity as compared to the degradation products of synthetic polymers (lactic acid in case of PLA) which leads to low immunogenicity (Rai et al., 2011; Basnett et al., 2018; Constantinides et al., 2018). This is because the hydroxyl acids such as hydroxybutyric acid, hydroxyoctanoic acid are natural metabolites. In addition, they degrade via surface degradation as opposed to the bulk degradation observed in the case of PLA, resulting in a controlled degradation of the construct. Hence PHAs are advantageous over synthetic polymers in the context of biomedical applications (Volova, 2004; Rai et al., 2011). The absence of any non-toxic compound produced during polymer degradation along with it being non-immunogenic enhances the acceptance of the scaffold by the body and hence promotes biocompatibility (Zhao et al., 2003; Zheng et al., 2005). PHAs have a diverse monomeric composition resulting in versatile physical properties making them applicable in a wide range of applications. Compared to the other natural polymers such as collagen, PHAs can be produced on a large scale, in a sustainable manner. Based on life cycle analysis, PHAs have been considered to be the safest options amongst the bioplastics that are currently available (Lakshmanan et al., 2013; Basnett et al., 2017). There are various *in vitro* and *in vivo* cytocompatibility studies conducted on PHA scaffolds which conclude that the scaffolds promoted angiogenesis, cell growth, and enabled cell attachment (Williams et al., 1999; Shishatskaya et al., 2002; Sevastianov et al., 2003; Qu et al., 2006; Shijun et al., 2016). The effect of PHA degradation products was established by Sun et al. (2007) where cellular responses of the mouse fibroblast cell line L929 were studied in the presence of PHA degradation products. It was observed that MCL-PHAs are even more biocompatible than the SCL-PHAs (Sun et al., 2007). Hence, Polyhydroxyalkanoates exhibit excellent biocompatibility and bioresorbability which make them highly suitable for their application in wound healing and other medical applications. The biodegradability rate of the PHAs depends on various factors such as the local environment, PHA composition, pH, moisture, surface area, microbial and enzymatic activity. PHA can be degraded by eukaryotic lipases and esterases in addition to environmental degradation (Ojumu et al., 2004). Volova et al. (2014) investigated the *in vivo* degradation of various PHA blends. They concluded that the biodegradability rate was higher in PHA copolymers with low crystallinity.

Artificial Intelligence (AI) algorithms represented by Deep Learning Neural Networks (Goodfellow et al., 2016) have shown huge impacts already in computer sciences and are expanding their applications to almost all human activities such as economy, science, engineering, psychology, linguistics, and more. In recent decades, these sudden jumps in advancements are realized through the exponential increases of computing power, the realization of massive parallel processing through Graphical Processing Units (GPU), and several important theoretical achievements. The theoretical achievements include finding Rectified Linear Unit (ReLU) (Lu et al., 2017) for feeding the gradient information more efficiently into the deeper layers of the neural network during the training of the algorithm and finding various important network structures such as Recurrent Neural Network (RNN), Long and Short Term Memory (LSTM), and Convolutional Neural Network (CNN). A thorough history of the AI developments can be found in Schmidhuber (2015). However, the robustness and the explainability (i.e., the ability for the mechanics of machine learning to be explained in human terms) of the decisions made by the AI algorithms are yet to be fully addressed (Szegedy et al., 2014; Holzinger et al., 2017). Also, it is observed that the algorithms would frequently show poor performance for unseen data (Mc Loone and Irwin, 2001), while requiring a huge amount of training data sets. Given these limitations of the current AI algorithms, there is still tremendous potential in the current AI algorithms to be applied to diverse areas to improve the performances of the overall outputs. This potential has started to gain impetus through the study of closed loop control systems in point of care devices (see Tan et al., 2019 for a recent review).

In this study we explore the use of a novel biopolymer material, an MCL-PHA, functionalized with graphene nanoplatelets (GnP) for the detection of the wound pathogen *P. aeruginosa*. Through the addition of graphene during the processing of the biopolymer into a wound dressing patch, we show that it is possible to detect the presence of pyocyanin. Pyocyanin is produced by almost all strains of *P. aeruginosa* during colonization of a wound and is hence a suitable diagnostic biomarker for infection. To the best of our knowledge, this is the first time that a Polyhydroxyalkanoate-based patch has been developed for the detection of pyocyanin. This study provides the potential for the development of a smart biocompatible wound dressing patch that could be used to detect the presence of an infection, followed by its reactive simultaneous treatment using a trained neural network to correct the errors in a prediction model. This will allow determination of the optimal release timing and concentration of the antimicrobial drug to be delivered in the wound bed, a truly revolutionary concept. Future work will build upon this *in vitro* study, focusing on the performance of the sensor in a real wound, *in vivo*, in order to determine and optimize the performance of the material as a sensor in this scenario.

## Materials and Methods

### Reagents and Media

#### Biopolymer Reagents

The MCL-PHA was produced using a Gram-negative bacterium *P. mendocina* CH50 which was obtained from the National Collection of Industrial and Marine Bacteria (NCIMB 10541), Aberdeen, United Kingdom. The chemicals for the production of MCL-PHA were purchased from Sigma-Aldrich or BDH Ltd. (Dorset, United Kingdom), VWR (Leicestershire, United Kingdom) unless otherwise stated. GnP were bought from Strem Chemicals with the dimensions of 6–8 nm thickness and 5 microns width.

#### Electrochemical Reagents

*Pseudomonas aeruginosa* reference strain PA14 (Mikkelsen et al., 2011) was used for all growth experiments. Luria Bertani (LB) media was used for all electrochemical measurements and growth experiments with *P. aeruginosa* PA14. LB was produced by mixing 10 g of tryptone (Fisher Scientific), 5 g NaCl (Sigma Aldrich), and 5 g yeast extract (Fluka) with 1000 mL of w/v dH_2_O. The media was then autoclaved at 121°C for 20 min. Iron (III) chloride (Sigma Aldrich) was mixed with dH_2_O to a concentration of 200 mM. Purified pyocyanin (Sigma Aldrich) was dissolved in EtOH to produce a stock concentration of 23.8 mM and was subsequently diluted in LB media to achieve the required working concentrations.

### Production and Characterization of MCL-PHA

Sterilized nutrient broth was inoculated using a single colony of *P. mendocina* CH50 and was incubated for 16 h at 30°C at 200 rpm. This was then used to inoculate the second stage media which was incubated at 30°C at 150 rpm until the optical density reached 1.6 without dilution. The second stage media comprised of minimal salt media (MSM) which included ammonium sulfate; 0.45 g/L, sodium hydrogen phosphate; 3.42 g/L and potassium dihydrogen phosphate; 2.38 g/L. Glucose at 20 g/L concentration was used as the carbon source. Magnesium sulfate heptahydrate and trace element were also added to the media at a concentration of 0.8 g/L and 1 mL/L, respectively. The inoculated second stage media was used to further inoculate the production media (10% of culture volume) for 48 h at 30°C at 150 rpm which comprised of MSM, glucose, magnesium sulfate heptahydrate and trace elements. The MSM comprised of ammonium sulfate; 0.50 g/L, sodium hydrogen phosphate; 3.80 g/L, potassium dihydrogen phosphate; 2.65 g/L.

After the incubation, the cells were harvested at 48 h by centrifugation at 4600 rpm for 30 min. The centrifuged cells were washed with distilled water followed by 10% ethanol and finally with distilled water. The obtained cells were homogenized for 15 min using a homogenizer. They were then kept at −20°C overnight and were finally placed in the freeze dryer for lyophilization.

The dried cells were used to extract polymer using the soxhlet extraction method. The cells were washed with methanol under reflux conditions for 24 h to remove impurities. Then the methanol was replaced with chloroform and the cells were incubated for 24 h under reflux conditions. This chloroform solution was used to extract polymer by concentrating it in a rotary evaporator. The polymer was then precipitated using ice-cold methanol solution. Although chloroform and methanol were used in this study, it should be noted that several non-chlorinated solvents such as cyclohexane, gamma-butyrolactone, butyl acetate and ethyl acetate have been used to extract PHAs (Aramvash et al., 2015; Jiang et al., 2018). Another environmentally sustainable method used in the extraction of PHAs include the use of supercritical fluid (Kunasundari and Sudesh, 2011).

#### Fourier Transform Infrared Spectroscopy (FTIR)

Chemical characterization of the polymer was carried out using by Attenuated Total Reflectance Fourier Transform Infrared (ATR-FTIR) spectroscopy. The analyses were performed in a spectral range of 4000–400 cm^–1^ with a resolution 4 cm^–1^ using PerkinElmer FTIR spectrometer Spectrum Two (PerkinElmer Inc., United States).

#### Molecular Weight Analysis

The number average molecular weight (M_n_), and the weight average molecular weight (M_w_) of the polymer were determined using gel permeation chromatography (GPC, Model 1260 Infinity GPC, Agilent Technologies) as described in Constantinides et al. (2018).

### Graphene-Biopolymer Composite Production

**FIGURE 1:**
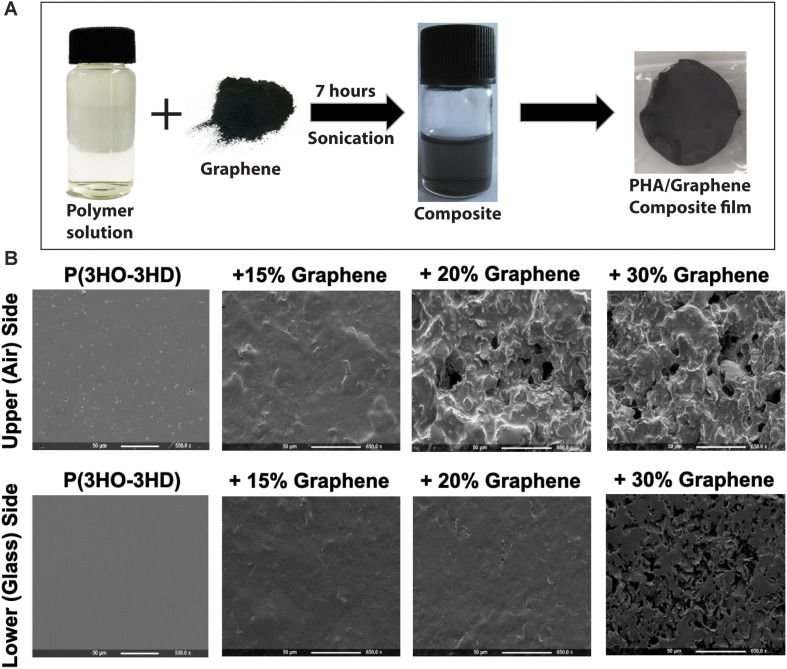
**(A)** The production of P(3HO-co-3HD)/graphene films, consisting of mixing the polymer solution with the graphene followed by casting in a glass petri dish. **(B)** Scanning electron microscopy of the film showing the change in the surface topography of the films with varying amounts of graphene content (both the upper side and the lower side of the films are shown).

The composite film was prepared using solvent casting by weighing 0.5 g of MCL-PHA and adding to 10 mL of chloroform. This solution was left stirring overnight until completely dissolved resulting in a 5 wt% solution of the polymer. Once the MCL-PHA was dissolved, different concentrations (15, 20, and 30%) of GnP were added. This was then vortexed for a minute and left to sonicate in a water bath sonicator for 7 h (Figure 1A). After sonication, the contents were immediately poured into a petri dish covered to allow slow and even evaporation and left to air dry in a fume cupboard. Upon drying, water was added in the petri dish and subsequently the composite films were peeled and air dried. The water is required because MCL-PHA is elastomeric in nature and tends to stick to the glass petri dish. Adding water to the petri dish helps in the removal of the film without causing a tear in the film.

### Cell Proliferation Study

A cell proliferation study was carried out to test the cytocompatibility of the composite films with the mammalian cells. It was carried out using HaCaT cells (keratinocyte). Cells at a seeding density of 10,000 cells/mL were added directly on to the sterile graphene composite film samples. They were cultured for 24 h. Cell viability was measured using Alamar Blue assay. TCP was used as a positive control. Neat MCL-PHA was tested for comparative study.

### Graphene-Polymer Surface Characterization

Scanning electron microscopy analysis was performed to evaluate the surface topography of the MCL-PHA/graphene composite films. The SEM images were taken using a beam of 5 keV at 10 cm working distance (JEOL 5610LV-SEM). For the analysis, all the samples were coated with gold for 2 min using a EMITECH-K550 gold spluttering device. This analysis was carried out at the Eastman Dental Hospital, University College London.

### Electrode Preparation and Chamber Design

Commercially available screen-printed carbon electrodes were purchased from DropSens. Standard carbon electrodes (DRP110), standard carbon modified with carbon nanotubes (DRP110-CNT), and standard carbon modified with graphene (DRP110-GPH) were used. Where the MCL-PHA was used as the sensing substrate, the counter electrode and reference electrode on a standard carbon electrode (DRP110) completed the electrochemical cell. In order to provide a stable reference, the silver reference electrode was modified by incubating the electrode surface for 10 min in a 200 mM solution of Iron(III) chloride in dH_2_O. It was found that 3 μl of Iron(III) chloride was sufficient to cover the reference electrode without touching the working or counter electrodes. Following the addition of the chloride, the electrodes were thoroughly rinsed in distilled water.

**FIGURE 2:**
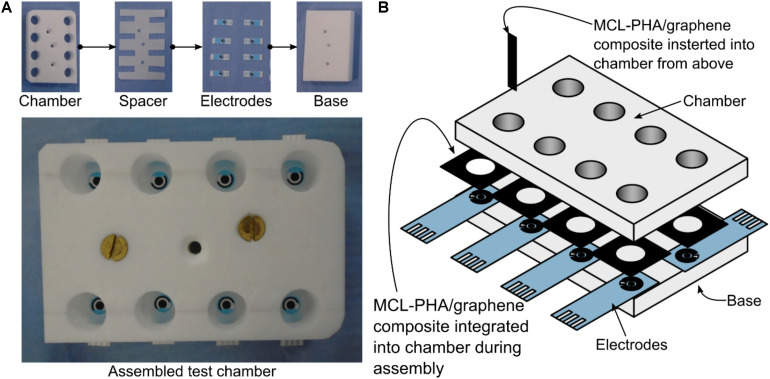
Bespoke electrode chambers used for measurements with commercial electrodes and MCL-PHA/graphene composite. The chambers were cut from a 15-mm-thick sheet of PTFE and assembled using silicon adhesive sealant. 1 mL of solution was used in each chamber. **(A)** Photographs of the layers of the assembly. **(B)** A detailed view of the assembly, showing the two locations where the PHA/graphene composite was inserted.

Electrode chambers were produced through the use of a custom-made PTFE plate (Figure 2A). The 15-mm-thick plate consisted of eight 16-mm-diameter chambers, a base layer of PTFE and a leveling layer to accommodate the thickness of the electrodes. The top plate was mounted on top of the electrodes and the bottom plate using silicon adhesive sealant and allowed to cure for 24 h prior to use. The MCL-PHA/graphene composite was cut into 5 mm strips with a sharp scalpel and held in the prepared chamber with a pair of metal forceps, so that approximately 5 mm of the MCL-PHA/graphene composite was submerged in the media (taking care that the forceps were not in contact with the media). Alternatively, the MCL-PHA/composite was integrated into the bottom of the electrode chamber by cutting a 10-mm-diameter hole through the center. This allowed the biopolymer patch to be integrated over the top of the commercial electrode (Figure 2B). 1 mL of LB was used in the electrode chambers during the experiments.

### MCL-PHA/Graphene Composite Patch Conductivity Measurements

The complex impedance of the polymers was measured using a PalmSens3 potentiostat (PalmSens) in a two-electrode configuration (Working – Counter/Reference). Two gold electrodes with 1 mm separation were brought into contact with the MCL-PHA/graphene composite patch, where a 0.5 V_DC_ with a 0.25 V_AC_ signal amplitude was applied across the frequency range from 0.1 to 10 kHz. In total, 51 frequency points were measured on a logarithmic scale. The reference measurement was performed by measuring the impedance between the two gold electrodes in free space. Both the smooth and rough surfaces (bottom- and top-side) of the MCL-PHA/graphene composite patches were measured, with three randomly positioned measurements, from which an average value was recorded.

### Electrochemical Measurements

Square wave voltammetry (SWV) was used to perform all electrochemical measurements, based upon previously published parameters for the detection of pyocyanin (Sharp et al., 2010). A potential window from −600 to 400 mV (vs. Ag/AgCl) was used, with a step potential of 2 mV and an amplitude of 50 mV. The measurement frequency used was 25 Hz. Electrochemical sweep data was either recorded in PSTrace, or directly in Matlab, by controlling the potentiostat through the PalmSens software development kit^1^.

### Standard Curve Measurements

Pyocyanin was diluted in sterile LB media to produce a working stock with a starting concentration of 2.5 mM. 1 mL of sterile LB was added to each electrode chamber and an initial SWV measurement was performed. Following this, 2 μl of pyocyanin working stock was added to the chamber and mixed by aspiration using the pipette, to achieve a final pyocyanin concentration of 5 μM. An SWV measurement was then performed and a further 2 μl of pyocyanin working stock was added in order to increase the concentration to 10 μM pyocyanin, followed by a further SWV measurement. This was repeated until a final calculated concentration of 97 μM pyocyanin existed in the measurement chamber.

### *P. aeruginosa* Measurements

*Pseudomonas aeruginosa* PA14 was used to produce cultures by inoculating 2 mL of sterile LB in a 30 mL universal bottle with a single colony of PA14 from a LB solid agar plate. This was then incubated at 37°C in a shaking incubator for 24 h. The overnight culture was then added to the electrode chamber and SWV measurements were performed with the biopolymer or commercial electrodes as described above.

## Results and Discussion

### Biopolymer Production With Integration of Graphene Platelets and Cell Proliferation Study

Medium chain length Polyhydroxyalkanoate was produced by *P. mendocina* CH50 using glucose as the carbon source. ATR-FTIR was used to identify the purified polymer as an MCL-PHA. Characteristic peaks (1727.4 cm^–1^, corresponded to the ester carbonyl bond and 1161 cm^–1^ corresponded to the C-O stretching) of MCL-PHA were present in the FTIR spectrum (see Supplementary Data Sheet S1 – supplementary information). Molecular weight, M_w_, of the MCL-PHA was measured to be 542 kDa with a polydispersity index (PDI) of 3. The percentage cell viability on the neat biopolymer film at 24 h was 85%. On the composite films with 15, 20, and 30% graphene content, %cell viability were 55, 53, and 49% respectively at 24 h. There was no significant difference between the films. There was a decrease in the cell viability in comparison to the neat biopolymer film.

### Biopolymer Composite Surface Properties and Resistance Measurements on MCL-PHA/Graphene Composites

**FIGURE 3:**
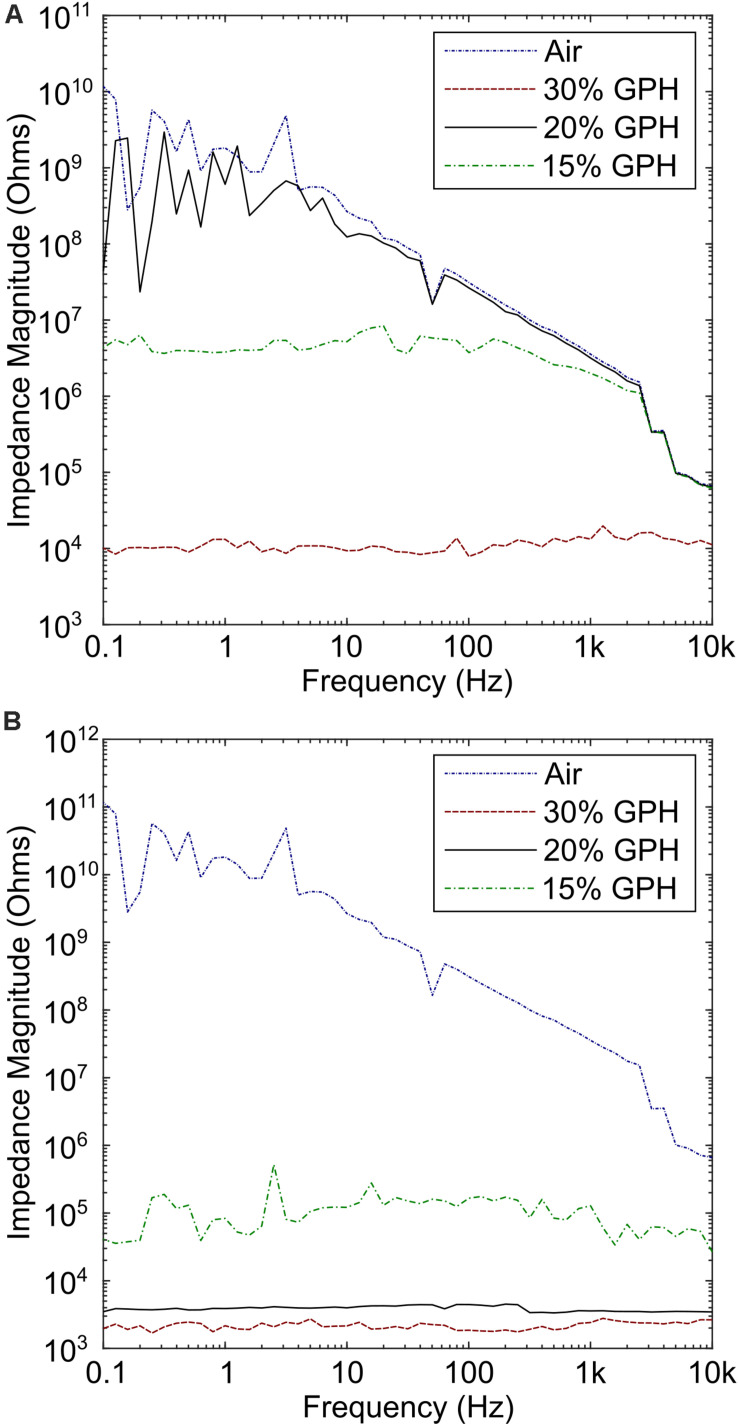
Conductivity measurements of the upper (air) side and lower (glass) side of the composite film. **(A)** High impedance was observed across all concentrations of graphene, with the lowest observed in the 30% graphene polymer. **(B)** Measurements performed on the lower side of the polymer indicate that the resistance is several times lower, particularly for the 30% graphene polymer.

SEM analysis of the two sides of the polymer film indicated different morphologies, which were influenced by the concentration of graphene added to the MCL-PHA and the side of the polymer (exposed to air or glass while casting in a glass petri dish) was being considered. As the concentration of graphene increased, both the upper and lower surfaces of the biopolymer increased in roughness (Figure 1B). Furthermore, initial electrochemical measurements with the MCL-PHA/graphene composites indicated that the response was dependent upon which side of the composite was used as a sensing surface. To understand the difference between the upper and lower surfaces, conductivity measurements were performed from 0.1 to 10 kHz. The measurements show that the rough, upper (air) side of the composite had a high resistance, regardless of the concentration of graphene present in the sample (Figure 3A). In contrast, it was found that the resistance of the lower (glass) side of the composite was several orders of magnitude less (Figure 3B). Furthermore, the resistance of the smooth lower side of the composite was found to be concentration dependent. As the concentration of graphene increased, the impedance of the layer decreased from a mean 111.8 kOhms (SD 79.4 kOhms) across the frequency range measured with the MCL-PHA/15 wt% graphene composite to 2.18 kOhms (SD 276 Ohms) for the MCL-PHA/30 wt% graphene composite. This suggests that during the curing phase of the MCL-PHA/graphene composite, the graphene settles toward the bottom of the polymer in a concentration dependent manner. Interestingly, the upper, rough surface of the 30 wt% MCL-PHA/graphene composite had a higher resistance than the rough surface with other concentrations of graphene. This is probably caused by the high graphene concentration in the 30 wt% composite, which settled down better toward the lower glass face. As this study is focused on the electrochemical properties and not the mechanical properties, the lower (glass) biopolymer side was used for subsequent electrochemical experiments as this side had the lowest resistance.

### Detection of Pyocyanin on Carbon-Based Substrates

**FIGURE 4:**
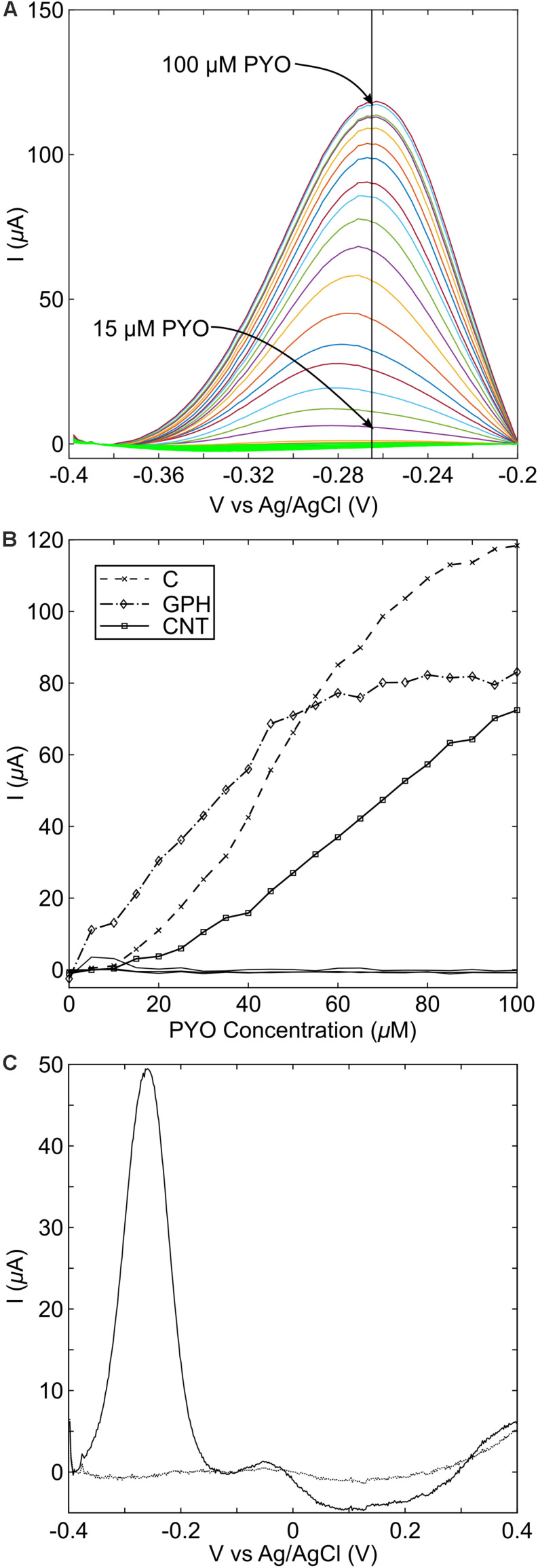
Pyocyanin standard curve measurements with different carbon electrode materials. **(A)** Peaks are easily identifiable following the addition of pyocyanin from 15 μM. On standard carbon screen printed electrodes, the peak was found to occur at –265 mV vs. Ag/AgCl; **(B)** Standard curve measurements at the peak potential for each measurement. C, standard carbon; GPH, graphene; CNT, carbon nanotubes; **(C)** overnight growth of PA14 shows that pyocyanin production can readily be detected on standard screen-printed carbon electrodes.

The use of graphene as a basis for the detection of pyocyanin relies on a redox reaction, which is linear across the range of interest. A series of standard curve experiments were performed to explore this, using screen printed carbon, graphene and carbon nanotube (CNT) electrodes, across a range of concentrations from 0 to 100 μM pyocyanin. A large background current was observed upon DPV measurements will all electrode types, therefore, a computer script was written in order to remove the background current, so that the peak could easily be resolved (Supplementary Data Sheet S2). In our study, across all types of carbon electrodes, a peak was observed between −265 and −292 mV vs. Ag-AgCl (Figure 4A), which is similar to SWV peaks previously reported for pyocyanin (Sharp et al., 2010; Bellin et al., 2014; Sismaet et al., 2016). For the graphene modified electrodes, the pyocyanin-current curve was linear up to 45 μM pyocyanin, at which point the rate of current increase changed (Figure 4B). In order to assess the implications of this for our sensor, we measured an overnight culture of *P. aeruginosa* PA14 after growth in conditions that promoted high concentrations of pyocyanin (Figure 4C). These measurements demonstrate that a redox peak relating to pyocyanin can be observed with a peak height of 49.36 μA, corresponding to approximately 42 μM pyocyanin, on the basis of the standard curve when a spline interpolation was used. In other studies (Wilson et al., 1988; Muller et al., 2009), clinically relevant concentrations of pyocyanin have been found to be less than 50 μM, highlighting that further development would be required to use these commercial screen printed electrodes as a sensor substrate for the linear detection of pyocyanin.

### Detection of Pyocyanin on the MCL-PHA/Graphene Composites With Different Concentrations of Graphene

**FIGURE 5:**
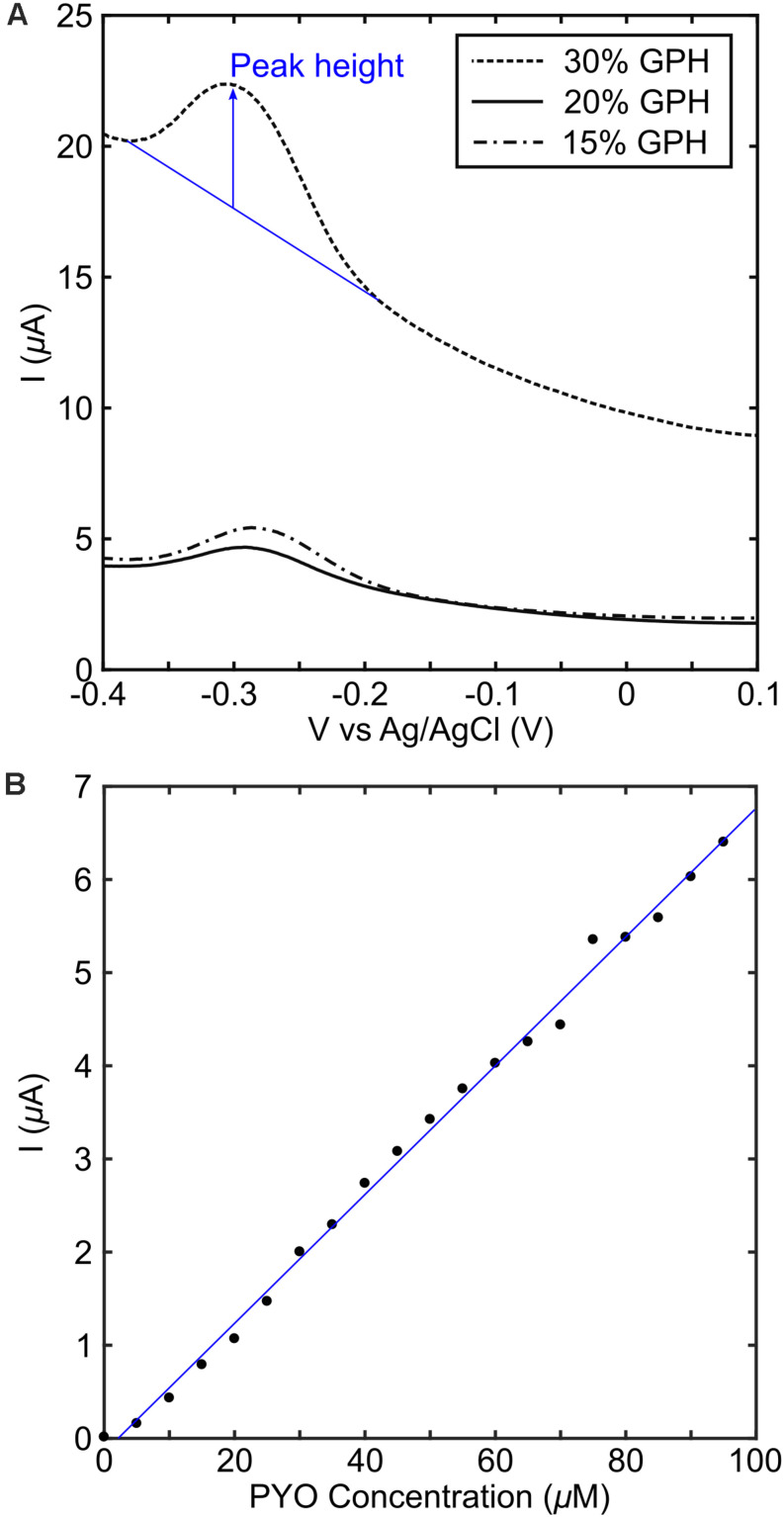
Electrochemical performance of MCL-PHA/graphene composites. **(A)** The composites with different concentrations of graphene were measured in a buffer of LB media containing 100 μM pyocyanin. The peak height (indicated in blue) was obtained using the approach described in SI1. **(B)** A standard curve was produced using the 30 wt/v% the PHA/graphene composite, with pyocyanin added in 5 μM steps up to 95 μM. The blue line represents a linear fit and was used to calculate pyocyanin concentration in the PA14 experiments.

The MCL-PHA/graphene composites with different concentrations of graphene were tested in conjunction with pyocyanin in order to determine the electrochemical performance, in contrast to the commercially available electrodes measured above. This was achieved by placing a strip of the MCL-PHA/graphene composite film, connected using forceps, into a well with a commercial screen-printed carbon electrode in the bottom. The screen-printed carbon electrode was used as the counter electrode and Ag-AgCl reference electrode, whereas the MCL-PHA/graphene composite formed the working electrode. The results indicate that a peak can be resolved in the SWV plot at −287 mV (Figure 5A), which is close to the location of the peak observed on graphene and CNT modified electrodes. This suggested that a similar electron transfer mechanism to that observed with the DRP110-GPH commercial electrodes was occurring. The 30% MCL-PHA/graphene composite showed the largest peak response to pyocyanin and was therefore explored further through the production of a standard curve (Figure 5B). This showed that the MCL-PHA/graphene composite has a lower current response to pyocyanin than the commercially modified electrodes. However, the response to increasing concentrations of pyocyanin is linear and peaks can be resolved from a low concentration of approximately 5 μM pyocyanin.

### Measurement of Pyocyanin With the MCL-PHA/Graphene Composite Electrodes

**FIGURE 6:**
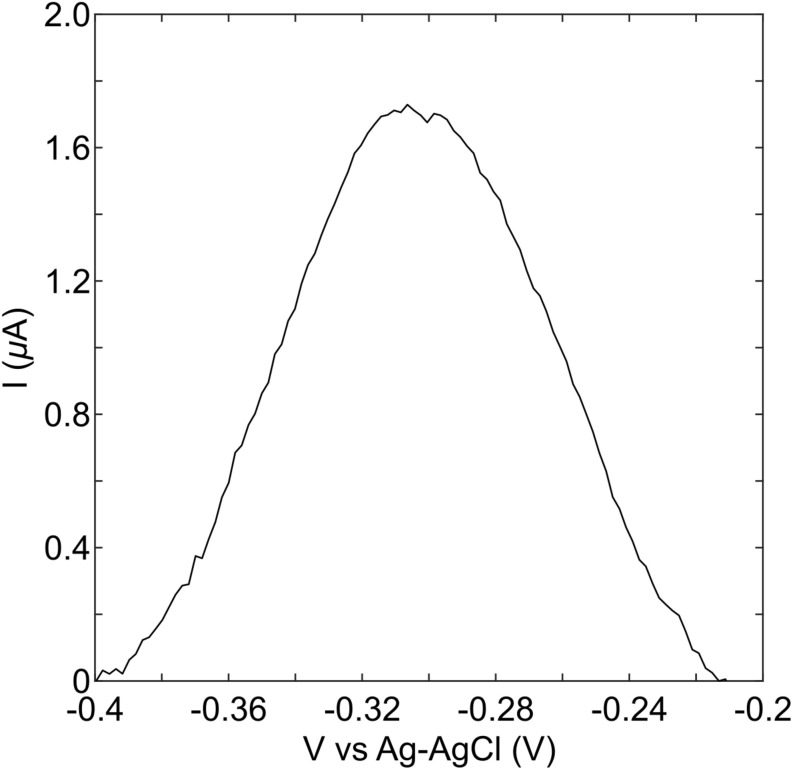
Detection of pyocyanin in an overnight culture of PA14. An aliquot of overnight culture was placed onto the graphene doped biopolymer and DPV measurements were performed. These show that a peak is observed in the same location as that seen in experiments with purified pyocyanin, suggesting that it is possible to detect the presence of pyocyanin in an overnight culture of PA14 using the biopolymer as a sensor.

The stationary phase culture of *P. aeruginosa* PA14 was strongly pigmented with a blue-green color, indicating the production of pyocyanin. Measurement of the overnight culture with a strip of the MCL-PHA/graphene composite indicated that a clear peak can be observed when PA14 is present (Figure 6). Much like the measurements performed using the commercial electrodes, these results confirmed that it is possible to identify the presence of *P. aeruginosa* PA14, once high concentrations of pyocyanin have been produced. Although the pyocyanin peak for the MCL-PHA/graphene composite was much lower than that observed with the commercial screen-printed electrodes, the results demonstrate the potential of the composite for the indirect identification of *P. aeruginosa* PA14.

In contrast to an overnight culture of *P. aeruginosa*, wound exudate is a complex matrix consisting of potential interferents including, glucose, pH, proteins, human cells, and other microorganisms. A previous study has demonstrated the ability to detect pyocyanin electrochemically in wound exudate with a carbon based electrode (Sismaet et al., 2016). In terms of polymicrobial infections, we have shown previously that it is possible to detect pyocyanin in a polymicrobial competition model in conjunction with *Staphylococcus aureus* (Ward et al., 2014). The production rate of pyocyanin in the presence of other common clinical isolates has also been investigated. In this study it was found that there was no statistical difference in pyocyanin concentration when *P. aeruginosa* was co-cultured alongside *Enterococcus faecalis*, *S. aureus*, or *Staphylococcus epidermidis* (Santiveri et al., 2018). Hence, literature suggests that it will be possible to electrochemically identify the presence of pyocyanin in wound exudate. In future studies, we will explore if this is also possible with our MCL/PHA graphene composite.

Electrochemical detection of pyocyanin has been explored in other studies with a view to producing low cost diagnostic tests. Sharp et al. (2010) demonstrate the ability to detect the presence of pyocyanin in concentration ranges between 1 and 100 μM using a carbon fiber tow in buffer. Alatraktchi et al. (2016) used commercially available screen printed gold electrodes to detect pyocyanin in a concentration range between 2 and 100 μM with cyclic voltammetry in buffer. This shows that the results reported in our study with the biocompatible polymer are comparable to other work. Interestingly, Ciui et al. (2018) produced a “swipeable” sensor, capable of detecting pyocyanin between 10 and 100 nM with a carbon electrode screen printed onto a disposable glove. This demonstrates that much higher sensitivities can be achieved with low cost, carbon-based substrates.

### Closed Loop Feedback and Control System Concept and Design

The MCL-PHA described in this paper has previously been demonstrated to be non-cytotoxic toward HaCaT cells (keratinocytes) *in vitro*. This study potentially adds additional benefits to the MCL-PHA by allowing it to be used as a sensing substrate to detect the presence of one potentially pathogenic microorganism. In order to fully exploit the benefits of MCL-PHA based graphene composites as a novel sensing circuit, we explored a feedback and control system that could be used to release antimicrobial agents into the wound bed when an infection is identified. Heavy metal ions, such as silver (Jung et al., 2008; Fromm, 2013; Sharma et al., 2015) have antimicrobial properties and could be electrochemically dissolved by applying a potential between two silver electrodes. This would cause silver ions to be dissolved into the wound bed and exert a toxic effect on the invading pathogen. Integration of a printed sliver substrate within the wound dressing could therefore provide a mechanism through which an antimicrobial metal ion could be electrochemically released on demand, as soon as pyocyanin is detected. The approach is conceptually attractive as the same electrochemical instrumentation used for the measurement could also be used to release silver ions from the silver substrate.

**FIGURE 7:**
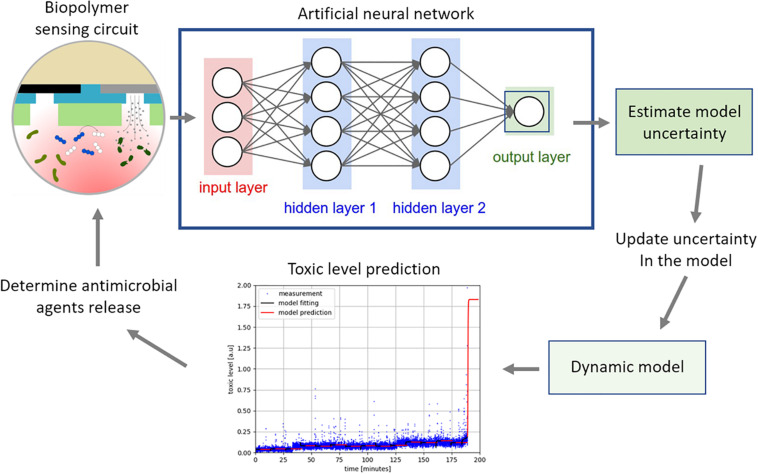
The measurement from the electrochemical biopolymer sensor are used as the input to the neural network, *a priori* trained, to estimate the model uncertainty. The uncertainty in the dynamic model is updated to improve the prediction accuracy of the model. The model predicts changes in the pyocyanin concentration as shown in the graph, where the blue is measured data and the red is the prediction. When the pyocyanin increases above a threshold level, another algorithm would determine optimal time and concentration of the antimicrobial silver required to kill *P. aeruginosa*.

To achieve this, we designed an overall structure of the feedback control system for regulating the concentration of silver to a toxic level while maintaining the concentration at a minimum used as shown in Figure 7. The neural network is trained off-line using simulated data, generated using the dynamic model represented in a set of ordinary differential equations (Dockery and Keener, 2001), to estimate the uncertainties. Training the network using simulated data is common practice when there is limited real measurement data available to train the network. The trained network determines the model uncertainty using the measurement from the sensing circuit. The dynamic model predicts the future toxic level after correcting the model error to identify whether the toxic level was achieved or not. The graph in Figure 7 demonstrates the prediction capability of the algorithm where the blue lines are the true toxic level and the red is the predicted one. This could be used to determine when and how much of the antimicrobial agents should be released to exert a toxic effect. Note that the true toxic level is generated using a stochastic molecular interaction model in Barbuti et al. (2008). It is our aim to build and verify this model further in the future through the production of in-depth experimental data.

## Conclusion

This paper describes, for the first time, a novel MCL-PHA/graphene composite wound dressing patch that can be used to detect the presence of an infection by *P. aeruginosa* PA14, a major wound pathogen in real time. The study demonstrated that clinically relevant concentrations of pyocyanin can be reliably detected using the composite. Furthermore, an outline closed loop algorithm was outlined and will be developed further to create a system which could be used to dynamically control silver concentrations within a wound bed to actively kill *P. aeruginosa* in response to an infection. In future work, we will explore the use of the developed composite to detect the presence of *P. aeruginosa* PA14 in complex clinical samples along with the optimization of the biopolymer composite properties for an efficient and effective smart wound dressing.

## Data Availability Statement

All datasets presented in this study are included in the article/Supplementary Material. Data used to create key tables is provided in Supplementary Table S1.

## Author Contributions

All authors contributed to the design and conception of the study, manuscript revision, read and approved the submitted version. AW, DC, and GN carried out electrochemical measurements. PB, PD, and IR produced the MCL-PHA using bacterial fermentation and the MCL-PHA/graphene composite. CR and SC performed the conductivity measurements. JK produced the control and feedback model.

## Conflict of Interest

The authors declare that the research was conducted in the absence of any commercial or financial relationships that could be construed as a potential conflict of interest.

## Footnotes

1 www.palmsens.com

## References

[B1] Alatraktchi F. A. Breum Andersen S. Krogh Johansen H. Molin S. Svendsen W. E. (2016). Fast selective detection of pyocyanin using cyclic voltammetry. *Sensors* 16:408. 10.3390/s16030408 27007376 PMC4813983

[B2] Aramvash A. Gholami-Banadkuki N. Moazzeni-Zavareh F. Hajizadeh-Turchi S. (2015). An environmentally friendly and efficient method for extraction of PHB biopolymer with non-halogenated solvents. *J. Microbiol. Biotechnol.* 25 1936–1943. 10.4014/jmb.1505.05053 26198125

[B3] Barbuti R. Caravagna G. Maggiolo-Schettini A. Milazzo P. Pardini G. (2008). “The calculus of looping sequences,” in *Formal Methods for Computational Systems Biology*, eds Bernardo M. Degano P. Zavattaro G. (Berlin: Springer), 387–423. 10.1007/978-3-540-68894-5_11

[B4] Basnett P. Lukasiewicz B. Marcello E. Gura H. K. Knowles J. C. Roy I. (2017). Production of a novel medium chain length poly(3-hydroxyalkanoate) using unprocessed biodiesel waste and its evaluation as a tissue engineering scaffold. *Microb. Biotechnol.* 10 1384–1399. 10.1111/1751-7915.12782 28905518 PMC5658593

[B5] Basnett P. Marcello E. Lukasiewicz B. Panchal B. Nigmatullin R. Knowles J. C. (2018). Biosynthesis and characterization of a novel, biocompatible medium chain length polyhydroxyalkanoate by *Pseudomonas* mendocina CH50 using coconut oil as the carbon source. *J. Mater. Sci. Mater. Med.* 29:179. 10.1007/s10856-018-6183-9 30506294

[B6] Bellin D. L. Sakhtah H. Rosenstein J. K. Levine P. M. Thimot J. Emmett K. (2014). Integrated circuit-based electrochemical sensor for spatially resolved detection of redox-active metabolites in biofilms. *Nat. Commun.* 5:3256. 10.1038/ncomms4256 24510163 PMC3969851

[B7] Bodey G. P. Bolivar R. Fainstein V. Jadeja L. (1983). Infections caused by *Pseudomonas aeruginosa*. *Rev. Infect. Dis.* 5 279–313. 10.1093/clinids/5.2.279 6405475

[B8] Byrom D. (1994). *Plastics From Microbes: Microbial Synthesis of Polymers and Polymer Precursors.* Munich: Hanser, 5–33.

[B9] Ciui B. Tertiş M. Cernat A. Sãndulescu R. Wang J. Cristea C. (2018). Finger-based printed sensors integrated on a glove for on-site screening of *Pseudomonas aeruginosa* virulence factors. *Anal. Chem.* 90 7761–7768. 10.1021/acs.analchem.8b01915 29851349

[B10] Constantinides C. Basnett P. Lukasiewicz B. Carnicer R. Swider E. Majid Q. A. (2018). In vivo tracking and 1H/19F magnetic resonance imaging of biodegradable Polyhydroxyalkanoate/Polycaprolactone blend scaffolds seeded with labeled cardiac stem cells. *ACS Appl. Mater. Interf.* 10 25056–25068. 10.1021/acsami.8b06096 29965724 PMC6338235

[B11] Dietrich L. E. P. Teal T. K. Price-Whelan A. Newman D. K. (2008). Redox-active antibiotics control gene expression and community behavior in divergent bacteria. *Science* 321 1203–1206. 10.1126/science.1160619 18755976 PMC2745639

[B12] Dockery J. D. Keener J. P. (2001). A mathematical model for quorum sensing in *Pseudomonas aeruginosa*. *Bull. Math. Biol.* 63:95. 10.1006/bulm.2000.0205 11146885

[B13] Dufresne A. Vincendon M. (2000). Poly (3-hydroxybutyrate) and poly (3-hydroxyoctanoate) blends: morphology and mechanical behavior. *Macromolecules* 33 2998–3008. 10.1021/ma991854a

[B14] Fromm K. M. (2013). Silver coordination compounds with antimicrobial properties. *Appl. Organometal. Chem.* 27 683–687. 10.1002/aoc.3047

[B15] Frykberg R. G. Banks J. (2015). Challenges in the treatment of chronic wounds. *Adv. Wound Care* 4 560–582. 10.1089/wound.2015.0635 26339534 PMC4528992

[B16] Gjødsbøl K. Christensen J. J. Karlsmark T. Jørgensen B. Klein B. M. Krogfelt K. A. (2006). Multiple bacterial species reside in chronic wounds: a longitudinal study. *Intern. Wound J.* 3 225–231. 10.1111/j.1742-481X.2006.00159.x 16984578 PMC7951738

[B17] Goodfellow I. Bengio Y. Courville A. (2016). *Deep Learning.* Cambridge, MA: The MIT Press.

[B18] Holzinger A. Biemann C. Pattichis C. S. Kell D. B. (2017). What do we need to build explainable AI systems for the medical domain?. *arXiv* [Preprint], Available online at: http://arxiv.org/abs/1712.09923 (accessed June 18, 2020).

[B19] Hyakutake M. Saito Y. Tomizawa S. Mizuno K. Tsuge T. (2011). Polyhydroxyalkanoate (PHA) synthesis by class IV PHA synthases employing Ralstonia eutropha PHB- 4 as host strain. *Biosci. Biotechnol. Biochem.* 75 1615–1617. 10.1271/bbb.110229 21821924

[B20] Jiang G. Johnston B. Townrow D. E. Radecka I. Koller M. Chaber P. (2018). Biomass extraction using non-chlorinated solvents for biocompatibility improvement of polyhydroxyalkanoates. *Polymers* 10:e070731. 10.3390/polym10070731 30960656 PMC6403533

[B21] Jung W. K. Koo H. C. Kim K. W. Shin S. Kim S. H. Park Y. H. (2008). Antibacterial activity and mechanism of action of the silver ion in *Staphylococcus aureus* and *Escherichia coli*. *Appl. Environ. Microbiol.* 74 2171–2178. 10.1128/AEM.02001-07 18245232 PMC2292600

[B22] Kirketerp-Møller K. Jensen P. Ø Fazli M. Madsen K. G. Pedersen J. Moser C. (2008). Distribution, organization, and ecology of bacteria in chronic wounds. *J. Clin. Microbiol.* 46 2717–2722. 10.1128/JCM.00501-08 18508940 PMC2519454

[B23] Kunasundari B. Sudesh K. (2011). Isolation and recovery of microbial polyhydroxyalkanoates. *Express Polym. Lett.* 5 620–634. 10.3144/expresspolymlett.2011.60

[B24] Kuo S.-H. Shen C.-J. Shen C.-F. Cheng C.-M. (2020). Role of pH value in clinically relevant diagnosis. *Diagnostics* 10:107. 10.3390/diagnostics10020107 32079129 PMC7167948

[B25] Lakshmanan R. Krishnan U. M. Sethuraman S. (2013). Polymeric scaffold aided stem cell therapeutics for cardiac muscle repair and regeneration. *Macromol. Biosci.* 13 1119–1134. 10.1002/mabi.201300223 23982911

[B26] Lau G. W. Hassett D. J. Ran H. Kong F. (2004). The role of pyocyanin in *Pseudomonas aeruginosa* infection. *Trends Mol. Med.* 10 599–606. 10.1016/j.molmed.2004.10.002 15567330

[B27] Lu Z. Pu H. Wang F. Hu Z. Wang L. (2017). “The expressive power of neural networks: a view from the width,” in *Proceedings of the 31st International Conference on Neural Information Processing Systems NIPS’17*, Long Beach, CA.

[B28] Mavrodi D. V. Blankenfeldt W. Thomashow L. S. (2006). Phenazine compounds in fluorescent *Pseudomonas* Spp. biosynthesis and regulation. *Annu. Rev. Phytopathol.* 44 417–445. 10.1146/annurev.phyto.44.013106.145710 16719720

[B29] Mc Loone S. Irwin G. (2001). Improving neural network training solutions using regularisation. *Neurocomputing* 37 71–90. 10.1016/S0925-2312(00)00314-3

[B30] McColl D. Cartlidge B. Connolly P. (2007). Real-time monitoring of moisture levels in wound dressings in vitro: an experimental study. *Intern. J. Surg.* 5 316–322. 10.1016/j.ijsu.2007.02.008 17499032

[B31] McColl D. MacDougall M. Watret L. Connolly P. (2009). Monitoring moisture without disturbing the wound dressing. *Wounds* 5 94–99.

[B32] McLister A. Mathur A. Davis J. (2017). Wound diagnostics: deploying electroanalytical strategies for point of care sensors and smart dressings. *Curr. Opin. Electrochem.* 3 40–45. 10.1016/j.coelec.2017.05.002

[B33] McManus A. T. Mason A. D. McManus W. F. Pruitt B. A. (1985). Twenty-five year review of*Pseudomonas aeruginosa* bacteremia in a burn center. *Eur. J Clin. Microbiol.* 4 219–223. 10.1007/BF02013601 3924612

[B34] Mikkelsen H. McMullan R. Filloux A. (2011). The *Pseudomonas aeruginosa* reference strain PA14 displays increased virulence due to a mutation in ladS. *PLoS One* 6:e29113. 10.1371/journal.pone.0029113 22216178 PMC3245244

[B35] Mostafalu P. Tamayol A. Rahimi R. Ochoa M. Khalilpour A. Kiaee G. (2018). Smart bandage for monitoring and treatment of chronic wounds. *Small* 14:1703509. 10.1002/smll.201703509 29978547

[B36] Muller M. Li Z. Maitz P. K. M. (2009). *Pseudomonas* pyocyanin inhibits wound repair by inducing premature cellular senescence: role for p38 mitogen-activated protein kinase. *Burns* 35 500–508. 10.1016/j.burns.2008.11.010 19286324

[B37] Nomura C. T. Taguchi S. (2007). PHA synthase engineering toward superbiocatalysts for custom-made biopolymers. *Appl. Microbiol. Biotechnol.* 73 969–979. 10.1007/s00253-006-0566-4 17123079

[B38] Ojumu T. V. Yu J. Solomon B. O. (2004). Production of Polyhydroxyalkanoates, a bacterial biodegradable polymers. *Afr. J. Biotechnol.* 3 18–24. 10.4314/ajb.v3i1.14910

[B39] Öncül O. Öksüz S. Acar A. Ülkür E. Turhan V. Uygur F. (2014). Nosocomial infection characteristics in a burn intensive care unit: analysis of an eleven-year active surveillance. *Burns* 40 835–841. 10.1016/j.burns.2013.11.003 24296064

[B40] Qu X. H. Wu Q. Liang J. Zou B. Chen G. Q. (2006). Effect of 3-hydroxyhexanoate content in poly (3-hydroxybutyrate-co-3-hydroxyhexanoate) on in vitro growth and differentiation of smooth muscle cells. *Biomaterials* 27 2944–2950. 10.1016/j.biomaterials.2006.01.013 16443271

[B41] Rai R. Keshavarz T. Roether J. A. Boccaccini A. R. Roy I. (2011). Medium chain length polyhydroxyalkanoates, promising new biomedical materials for the future. *Mater. Sci. Eng. R Rep.* 72 29–47. 10.1016/j.mser.2010.11.002

[B42] Reyes E. A. Bale M. J. Cannon W. H. Matsen J. M. (1981). Identification of *Pseudomonas aeruginosa* by pyocyanin production on Tech agar. *J. Clin. Microbiol.* 13 456–458. 10.1128/jcm.13.3.456-458.1981 6787067 PMC273813

[B43] Rezaie F. Momeni-Moghaddam M. Naderi-Meshkin H. (2019). Regeneration and repair of skin wounds: various strategies for treatment. *Intern. J. Lower Extrem. Wounds* 18 247–261. 10.1177/1534734619859214 31257948

[B44] Rivero G. Meuter M. Pepe A. Guevara M. G. Boccaccini A. R. Abraham G. A. (2020). Nanofibrous membranes as smart wound dressings that release antibiotics when an injury is infected. *Coll. Surf. A* 587:124313 10.1016/j.colsurfa.2019.124313

[B45] Santiveri C. R. Sismaet H. J. Kimani M. Goluch E. D. (2018). Electrochemical detection of *Pseudomonas aeruginosa* in polymicrobial environments. *Chem. Select* 3 2926–2930. 10.1002/slct.201800569

[B46] Scalamandré A. Bogie K. M. (2020). “Chapter 13 - smart technologies in wound prevention and care,” in *Innovations and Emerging Technologies in Wound Care*, ed. Gefen A. (Cambridge, MA: Academic Press), 225–244. 10.1016/B978-0-12-815028-3.00013-4

[B47] Schaarup C. Pape-Haugaard L. B. Hejlesen O. K. (2018). Models used in clinical decision support systems supporting healthcare professionals treating chronic wounds: systematic literature review. *JMIR Diabetes* 3 e11. 10.2196/diabetes.8316 30291078 PMC6238865

[B48] Schmidhuber J. (2015). Deep learning in neural networks: an overview. *Neural Netw.* 61 85–117. 10.1016/j.neunet.2014.09.003 25462637

[B49] Serra R. Grande R. Butrico L. Rossi A. Settimio U. F. Caroleo B. (2015). Chronic wound infections: the role of *Pseudomonas aeruginosa* and *Staphylococcus aureus*. *Expert Rev. Anti Infect. Ther.* 13 605–613. 10.1586/14787210.2015.1023291 25746414

[B50] Sevastianov V. I. Perova N. V. Shishatskaya E. I. Kalacheva G. S. Volova T. G. (2003). Production of purified polyhydroxyalkanoates (PHAs) for applications in contact with blood. *J. Biomater. Sci. Polym. Ed.* 14 1029–1042. 10.1163/156856203769231547 14661877

[B51] Sharma B. K. Saha A. Rahaman L. Bhattacharjee S. Tribedi P. (2015). Silver inhibits the biofilm formation of *Pseudomonas aeruginosa*. *Adv. Microbiol.* 05 677–685. 10.4236/aim.2015.510070

[B52] Sharma B. R. Harish D. Singh V. P. Bangar S. (2006). Septicemia as a cause of death in burns: an autopsy study. *Burns* 32 545–549. 10.1016/j.burns.2006.02.008 16797127

[B53] Sharp D. Gladstone P. Smith R. B. Forsythe S. Davis J. (2010). Approaching intelligent infection diagnostics: carbon fibre sensor for electrochemical pyocyanin detection. *Bioelectrochemistry* 77 114–119. 10.1016/j.bioelechem.2009.07.008 19666245

[B54] Sheets A. R. Hwang C. K. Herman I. M. (2016). *Developing “Smart” Point-of-Care Diagnostic Tools for “Next-Generation” Wound Care*, Chap. 17. ed. Laurence J. (Translating Regenerative Medicine to the Clinic: Academic Press), 251–264. Available online at: 10.1016/B978-0-12-800548-4.00017-6

[B55] Shijun X. Junsheng M. Jianqun Z. Ping B. (2016). *In vitro* three-dimensional coculturing poly3-hydroxybutyrate-co-3-hydroxyhexanoate with mouse-induced pluripotent stem cells for myocardial patch application. *J. Biomater. Appl.* 30 1273–1282. 10.1177/0885328215612115 26873635

[B56] Shishatskaya E. I. Volova T. G. Gitelson I. I. (2002). In vivo toxicological evaluation of polyhydroxyalkanoates. *Doklady Biol. Sci.* 383 109–111. 10.1023/a:1015325504494 12053558

[B57] Sismaet H. J. Banerjee A. McNish S. Choi Y. Torralba M. Lucas S. (2016). Electrochemical detection of *Pseudomonas* in wound exudate samples from patients with chronic wounds. *Wound Repair. Regen.* 24 366–372. 10.1111/wrr.12414 26815644 PMC4853203

[B58] Songkakul T. Bhushan P. Umasankar Y. Yokus M. Daniele M. A. Bhansali S. (2019). “Towards a long-term multi-site electrochemical wound monitoring system,” in *Proceedings of the 2019 IEEE SENSORS*, Montreal, QC.

[B59] Sun J. Dai Z. Zhao Y. Chen G. Q. (2007). *In vitro* effect of oligo-hydroxyalkanoates on the growth of mouse fibroblast cell line L929. *Biomaterials* 28 3896–3903. 10.1016/j.biomaterials.2007.05.011 17574664

[B60] Szegedy C. Zaremba W. Sutskever I. Bruna J. Erhan D. Goodfellow I. (2014). Intriguing properties of neural networks. *arXiv* [Preprint], Available online at: http://arxiv.org/abs/1312.6199 (accessed June 18, 2020).

[B61] Tan E. K. W. Au Y. Z. Moghaddam G. K. Occhipinti L. G. Lowe C. R. (2019). Towards closed-loop integration of point-of-care technologies. *Trends Biotechnol.* 37 775–788. 10.1016/j.tibtech.2018.12.004 30683459

[B62] Valappil S. P. Boccaccini A. R. Bucke C. Roy I. (2007). Polyhydroxyalkanoates in Gram-positive bacteria: insights from the genera *Bacillus* and *Streptomyces*. *Antonie Van Leeuwenhoek* 91 1–17. 10.1007/s10482-006-9095-5 17016742

[B63] Valappil S. P. Rai R. Bucke C. Roy I. (2008). Polyhydroxyalkanoate biosynthesis in *Bacillus cereus* SPV under varied limiting conditions and an insight into the biosynthetic genes involved. *J. Appl. Microbiol.* 104 1624–1635. 10.1111/j.1365-2672.2007.03678.x 18194257

[B64] Volova T. (2004). *Polyhydroxyalkanoates-Plastic Materials of the 21st Century: Production, Properties, Applications.* Hauppauge, NY: Nova Science Publishers.

[B65] Volova T. G. Shishatskaya E. I. Nikolaeva E. D. Sinskey A. J. (2014). In vivo study of 2D PHA matrices of different chemical compositions: tissue reactions and biodegradations. *Mater. Sci. Technol.* 30 549–557. 10.1179/1743284713Y.0000000470

[B66] Wang Y. Newman D. K. (2008). Redox reactions of phenazine antibiotics with ferric (Hydr)oxides and molecular oxygen. *Environ. Sci. Technol.* 42 2380–2386. 10.1021/es702290a 18504969 PMC2778262

[B67] Ward A. C. Connolly P. Tucker N. P. (2014). *Pseudomonas aeruginosa* can be detected in a polymicrobial competition model using impedance spectroscopy with a novel biosensor. *PLoS One* 9:e91732. 10.1371/journal.pone.0091732 24614411 PMC3948879

[B68] Williams S. Martin D. Horowitz D. Peoples O. (1999). PHA applications: addressing the price performance issue. I. Tissue engineering. *Int. J. Biol. Macromol.* 25 111–121. 10.1016/s0141-8130(99)00022-7 10416657

[B69] Wilson R. Sykes D. A. Watson D. Rutman A. Taylor G. W. Cole P. J. (1988). Measurement of *Pseudomonas aeruginosa* phenazine pigments in sputum and assessment of their contribution to sputum sol toxicity for respiratory epithelium. *Infect. Immun.* 56 2515–2517. 10.1128/iai.56.9.2515-2517.1988 3137173 PMC259599

[B70] Zhao K. Deng Y. Chen J. C. Chen G. Q. (2003). Polyhydroxyalkanoate (PHA) scaffolds with good mechanical properties and biocompatibility. *Biomaterials* 24 1041–1045. 10.1016/s0142-9612(02)00426-x 12504526

[B71] Zheng Z. Bei F. F. Tian H. L. Chen G. Q. (2005). Effects of crystallization of polyhydroxyalkanoate blend on surface physicochemical properties and interactions with rabbit articular cartilage chondrocytes. *Biomaterials* 26 3537–3548. 10.1016/j.biomaterials.2004.09.041 15621244

