# Supplementary material for: Toward a Closed Loop, Integrated Biocompatible Biopolymer Wound Dressing Patch for Detection and Prevention of Chronic Wound Infections
Source: Front Bioeng Biotechnol. 2020 Sep 1;8:1039. doi: 10.3389/fbioe.2020.01039 (PMC7493637; doi:10.3389/fbioe.2020.01039)
Supplement: Supplementary file 2 [file Data_Sheet_1.docx]

**Supplementary Information**

**Towards a closed loop, integrated biocompatible biopolymer wound dressing patch for detection and prevention of chronic wound infections**

Andrew C Ward^1^, Prachi Dubey^2^, Pooja Basnett^2^, Granit Lika^2^, Gwenyth Newman^1^, Damion K Corrigan^1^, Christopher Russell^4^, Jongrae Kim^5^, Samit Chakrabarty^4^, Patricia Connolly^1^, Ipsita Roy^3*^

^1^Department of Biomedical Engineering, University of Strathclyde, Glasgow.

^2^ School of Life Sciences, College of Liberal Arts and Sciences, University of Westminster, London

^3^ Department of Material Science and Engineering, Faculty of Engineering, University of Sheffield, Sheffield

^4^School of Biomedical Sciences, University of Leeds, Leeds, UK

^5^School of Mechanical Engineering, University of Leeds, Leeds, UK

* Corresponding Author:

Professor Ipsita Roy
I.Roy@sheffield.ac.uk

**Fourier Transform Infrared Spectroscopy (FTIR)**

The polymer produced was primarily characterised using FTIR to identify whether the polymer is an SCL-PHA or MCL-PHA. As shown from the Figure below, the spectral range was between 4000cm^-1^ to 400cm^-1^. There are three main peaks: First peak at approximately 2923cm^-1^ corresponds to a stretching CH_2_ or CH_3_ aliphatic group of the polymer. The second singular peak at 1727cm^-1^ correspond to C= O ester carbonyl group. The third main peak at 1161cm^-1^ correspond to a C-O stretching group. According to literature, these are the characteristic peak found in medium chain length polymers (Rai *et al*., 2011).


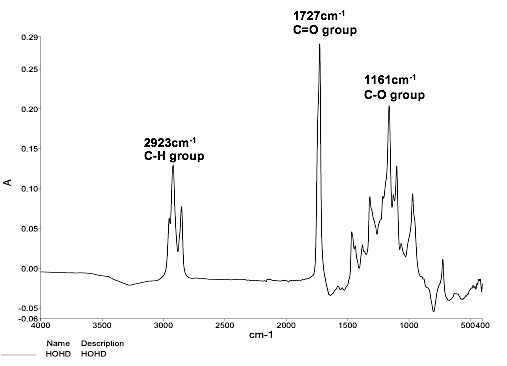


**Figure SI1: FTIR spectra of the MCL- PHA**
